# Supplementary material for: Nodosilinea signiensis sp. nov. (Leptolyngbyaceae, Synechococcales), a new terrestrial cyanobacterium isolated from mats collected on Signy Island, South Orkney Islands, Antarctica
Source: PLoS One. 2019 Nov 4;14(11):e0224395. doi: 10.1371/journal.pone.0224395 (PMC6827893; doi:10.1371/journal.pone.0224395)

PLOS ONE

Nodosilinea signiensis sp. nov. (Leptolyngbyaceae, Synechococcales), a new terrestrial cyanobacterium isolated from soil collected on Signy Island, South Orkney Islands, Antarctica

--Manuscript Draft--

**Manuscript Number:**	PONE-D-19-15186
**Article Type:**	Research Article
**Full Title:**	Nodosilinea signiensis sp. nov. (Leptolyngbyaceae, Synechococcales), a new terrestrial cyanobacterium isolated from soil collected on Signy Island, South Orkney Islands, Antarctica
**Short Title:**	Nodosilinea signiensis sp. nov. (Leptolyngbyaceae, Synechococcales), a new Antarctic cyanobacterium
**Corresponding Author:**	Faradina Merican, PhD<br>Universiti Sains Malaysia<br>Minden, Penang MALAYSIA
**Keywords:**	Leptolyngbyaceae, new species, 16S rDNA gene, Internal Transcribed Spacer ITS
**Abstract:**	Terrestrial cyanobacteria are very diverse and widely distributed in Antarctica, where they can form macroscopically visible biofilms on the surfaces of soils and rocks. We recently isolated several terrestrial cyanobacteria from soils collected on Signy Island, South Orkney Islands, Antarctica. Among them, we found a novel isolate of Nodosilinea, named here as Nodosilinea signiensis sp. nov. This new species is morphologically and genetically distinct from other described species. Morphological examination indicated that the new species is differentiated from others in the genus by cell size, cell shape, filament attenuation, sheath morphology and granulation. The 16S rDNA phylogenetic analyses clearly showed that N. signiensis is placed within the major clade of Nodosilinea and is distinct from other Nodosilinea sequences within the clade. The D1–D1´ helix of the 16S–23S ITS region of the new species was also different from previously described Nodosilinea species. This is the first report of a detailed characterization of a member of the genus Nodosilinea from Antarctica.
**Order of Authors:**	Faradina Merican, PhD
	Ranina Radzi
	Narongrit Muangmai
	Paul Broady
	Wan Maznah Wan Omar
	Peter Convey
**Opposed Reviewers:**	
**Additional Information:**	
**Question**	**Response**
**Financial Disclosure**<br><br>Enter a financial disclosure statement that describes the sources of funding for the work included in this submission. Review the submission guidelines for detailed requirements. View published research articles from *PLOS ONE* for specific examples.<br><br>This statement is required for submission	This study received funding support from YPASM Berth Support, YPASM Fellowship 304/PBIOLOGI/650963, RUI grant 1001/PBIOLOGI/811305 and Flagship grant 304/PBIOLOGI/650723/P131. Peter Convey is supported by NERC core funding to the BAS 'Biodiversity, Evolution and Adaptation' Team.

Powered by Editorial Manager® and ProduXion Manager® from Aries Systems Corporation

and **will appear in the published article** if the submission is accepted. Please make sure it is accurate.<br><br>**Unfunded studies**<br>Enter: *The author(s) received no specific funding for this work.*<br><br>**Funded studies**<br>Enter a statement with the following details:<br>• Initials of the authors who received each award<br>• Grant numbers awarded to each author<br>• The full name of each funder<br>• URL of each funder website<br>• Did the sponsors or funders play any role in the study design, data collection and analysis, decision to publish, or preparation of the manuscript?<br>• **NO** - Include this sentence at the end of your statement: *The funders had no role in study design, data collection and analysis, decision to publish, or preparation of the manuscript.*<br>• **YES** - Specify the role(s) played.<br><br>* typeset	
**Competing Interests**<br><br>Use the instructions below to enter a competing interest statement for this submission. On behalf of all authors, disclose any competing interests that could be perceived to bias this work—acknowledging all financial support and any other relevant financial or non-financial competing interests.<br><br>This statement **will appear in the published article** if the submission is accepted. Please make sure it is accurate. View published research articles from *PLOS ONE* for specific examples.	The authors have declared that no competing interests exist

**NO authors have competing interests**<br><br>Enter: *The authors have declared that no competing interests exist.*<br><br>**Authors with competing interests**<br><br>Enter competing interest details beginning with this statement:<br><br>*I have read the journal's policy and the authors of this manuscript have the following competing interests: [insert competing interests here]*<br><br>* typeset	
**Ethics Statement**<br><br>Enter an ethics statement for this submission. This statement is required if the study involved:<br><br>• Human participants<br>• Human specimens or tissue<br>• Vertebrate animals or cephalopods<br>• Vertebrate embryos or tissues<br>• Field research<br><br>Write "N/A" if the submission does not require an ethics statement.<br><br>General guidance is provided below. Consult the submission guidelines for detailed instructions. **Make sure that all information entered here is included in the Methods section of the manuscript.**	N/A

**Format for specific study types**<br><br>**Human Subject Research (involving human participants and/or tissue)**<br>• Give the name of the institutional review board or ethics committee that approved the study<br>• Include the approval number and/or a statement indicating approval of this research<br>• Indicate the form of consent obtained (written/oral) or the reason that consent was not obtained (e.g. the data were analyzed anonymously)<br><br>**Animal Research (involving vertebrate animals, embryos or tissues)**<br>• Provide the name of the Institutional Animal Care and Use Committee (IACUC) or other relevant ethics board that reviewed the study protocol, and indicate whether they approved this research or granted a formal waiver of ethical approval<br>• Include an approval number if one was obtained<br>• If the study involved *non-human primates*, add *additional details* about animal welfare and steps taken to ameliorate suffering<br>• If anesthesia, euthanasia, or any kind of animal sacrifice is part of the study, include briefly which substances and/or methods were applied<br><br>**Field Research**<br><br>Include the following details if this study involves the collection of plant, animal, or other materials from a natural setting:<br>• Field permit number<br>• Name of the institution or relevant body that granted permission	
**Data Availability**<br><br>Authors are required to make all data underlying the findings described fully available, without restriction, and from the time of publication. PLOS allows rare exceptions to address legal and ethical concerns. See the PLOS Data Policy and FAQ for detailed information.	Yes - all data are fully available without restriction

Powered by Editorial Manager® and ProduXion Manager® from Aries Systems Corporation

A Data Availability Statement describing where the data can be found is required at submission. Your answers to this question constitute the Data Availability Statement and **will be published in the article**, if accepted.<br><br>**Important:** Stating 'data available on request from the author' is not sufficient. If your data are only available upon request, select 'No' for the first question and explain your exceptional situation in the text box.<br><br>Do the authors confirm that all data underlying the findings described in their manuscript are fully available without restriction?	
**Describe where the data may be found in full sentences. If you are copying our sample text, replace any instances of XXX with the appropriate details.**<br><br>• If the data are **held or will be held in a public repository**, include URLs, accession numbers or DOIs. If this information will only be available after acceptance, indicate this by ticking the box below. For example: *All XXX files are available from the XXX database (accession number(s) XXX, XXX.)*.<br>• If the data are all contained **within the manuscript and/or Supporting Information files**, enter the following: *All relevant data are within the manuscript and its Supporting Information files.*<br>• If neither of these applies but you are able to provide **details of access elsewhere**, with or without limitations, please do so. For example:<br><br>*Data cannot be shared publicly because of [XXX]. Data are available from the XXX Institutional Data Access / Ethics Committee (contact via XXX) for researchers who meet the criteria for access to confidential data.*<br><br>*The data underlying the results presented in the study are available from (include the name of the third party*	All relevant data are within the manuscript files.

and contact information or URL).<br>• This text is appropriate if the data are owned by a third party and authors do not have permission to share the data.<br><br>* typeset	
Additional data availability information:	

Dear Editor,

We would like to submit the manuscript entitled ‘*Nodosilinea signiensis* sp. nov. (Leptolyngbyaceae, Synechococcales), a new terrestrial cyanobacterium isolated from soil collected in Signy Island, South Orkney Islands, Antarctica’ to be considered for publication in PLOS ONE.

Molecular phylogenetics has had a great impact on cyanobacterial taxonomy and many new species have been derived based on the new classification system. Here, we present the first detailed characterization combining the traditional and molecular approaches of a member from the genus *Nodosilinea* in an Antarctic environment. We believe this finding will be of interest to researchers and readers of your journal.

We declare that this manuscript is original, has not been published before and is not currently being considered for publication elsewhere.

We know of no conflicts of interest associated with this publication. As Corresponding Author, I confirm that the manuscript has been read and approved for submission by all the named authors.

For further enquiries regarding the manuscripts kindly address all correspondence to me at faradina@usm.my. I sincerely hope that you would find the work interesting and would consider it for publication in your journal. Thanking you in advanced.

Best regards,

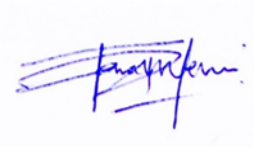

Faradina Merican
School of Biological Sciences,
Universiti Sains Malaysia

Manuscript
Click here to download Manuscript Nodosilinea signiensis Antarctica.docx

Nodosilinea signiensis sp. nov. (Leptolyngbyaceae, Synechococcales), a new terrestrial cyanobacterium isolated from soil collected on Signy Island, South Orkney Islands, Antarctica

Ranina Radzi[1], Narongrit Muangmai[2], Paul Broady[3], Wan Maznah Wan Omar[1], Peter Convey[4], Faradina Merican[1]*

[1]School of Biological Science, Universiti Sains Malaysia, Minden, Penang, Malaysia.

[2]Faculty of Fisheries, Kasetsart University, Chatuchak, Bangkok, Thailand.

[3]School of Biological Sciences, University of Canterbury, Christchurch, New Zealand.

[4]British Antarctic Survey, Cambridge, United Kingdom.

*Corresponding author

E-mail: faradina@usm.my

Abstract

Terrestrial cyanobacteria are very diverse and widely distributed in Antarctica, where they can form macroscopically visible biofilms on the surfaces of soils and rocks. We recently isolated several terrestrial cyanobacteria from soils collected on Signy Island, South Orkney Islands, Antarctica. Among them, we found a novel isolate of *Nodosilinea*, named here as *Nodosilinea signiensis* sp. nov. This new species is morphologically and genetically distinct from other described species. Morphological examination indicated that the new species is differentiated from others in the genus by cell size, cell shape, filament attenuation, sheath morphology and granulation. The 16S rDNA phylogenetic analyses clearly showed that *N. signiensis* is placed within the major clade of *Nodosilinea* and is distinct from other *Nodosilinea* sequences within the clade. The D1–D1´ helix of the 16S–23S ITS region of the new species was also different from previously described *Nodosilinea* species. This is the first report of a detailed characterization of a member of the genus *Nodosilinea* from Antarctica.

Introduction

Cyanobacteria are a widely distributed group of oxygenic photosynthetic prokaryotes that possess chlorophyll *a* and phycobiliproteins [1]. Despite their widespread occurrence and ecological importance, the taxonomy of cyanobacteria remains problematic. Cyanobacterial classification has recently undergone rapid revision based on the use of polyphasic approaches to define new taxa [2]. These approaches combine molecular characterization with cytomorphological and ecological characteristics and have been used ~~both in the erection of~~ new taxa and the validation of classically described taxa [3, 4].

Amongst the different cyanobacteria groups, Leptolyngbyaceae (Synechococcales) is one of the most taxonomically challenging. Representatives of this family are morphologically simple, often with ambiguous and indistinct morphological features that lead to uncertainty in identification [5]. However, species within the group can be distinguished based on molecular evaluation. Ongoing taxonomic revisionary work has resulted in over 21 new genera being erected from the original genus *Leptolyngbya*. Most of these are listed in various literature [6 - 11] and others are described [12 - 23].

The genus *Nodosilinea* [8] shows a clear phylogenetic separation from *Leptolyngbya* based on molecular assessment. The genus has been reported to possess a unique capability to form short convoluted lengths of trichomes within the bounding sheath, termed nodules. This was first documented in *Leptolyngbya nodulosa* [24]. The generic assignment of *L. nodolusa* was subsequently revised with the support of molecular phylogenetics and the species is now *Nodosilinea nodulosa* [24], the first member of the new genus. Subsequent studies have reported the ability to form nodules in all members of the genus when exposed to low-light conditions of < 4-μmol m^{-2} s^{-1} for 4 weeks. Some species have also been reported to perform nitrogen fixation [8, 24, 25]. Apart from nodule formation, the characteristics of the genus

resemble those of *Leptolyngbya* with uniseriate trichomes ranging from 0.5 to 3.5 µm wide within a thin sheath, similar vegetative and apical cell shapes, constriction at each cross wall and granules [26].

Representatives of the genus have been recorded from various habitats. *Nodosilinea* sp. LEGE 13457 and *Nodosilinea* sp. TM-3.1 were both isolated from the McMurdo Dry Valleys, Antarctica [27]. *N. ramsarensis* and *N. radiophila* were recorded from a thermal spring in Iran [28] and *N. nodulosa* from marine phytoplankton in the China Sea [24]. Other species are subaerial on walls (*N. epilithica* [8], *N. chupicuarensis* [25]) and occur in soils (*N. conica* [8]) and freshwater (*N. bijugata* [8, 29]).

In Antarctica, cyanobacteria are the most important primary colonizers of soils [30]. They play vital roles in soil stabilisation, photosynthetic carbon fixation, and the release of fixed nitrogen, whilst forming the base of the terrestrial food web [31]. Cyanobacteria are found across all geographical regions of Antarctica where they can form macroscopically visible mats, crusts or thin biofilms on the surfaces of soils and rocks and in streams, ponds and lakes, as well as occupying endolithic niches [32, 33].

Leptolyngbya is one of the most widely recorded genera in Antarctica [34 - 41]. However, detailed characterization combining morphological, ultrastructural and molecular approaches to date regognised only three species (*L. bijugata* [43], *L. borchgrevinkii* and *L. frigida* [44]) and described an additional two unidentified species [42].

Three species of *Leptolyngbya* (*L. foveolarum, L. notata* and *L. perelegans*) were recorded in a broad-scale floristic survey conducted on Signy Island, South Orkney Islands [34]. These classifications were based solely on morphological traits and have not subsequently been subjected to molecular assessment. In this study, we report the polyphasic characterization of a cyanobacterial strain superficially resembling *Leptolyngbya*, recently isolated from soils collected on Signy Island.

Materials and Methods

Sample origin and culture conditions

Mat samples were obtained from cracks and crevices in rocks and beneath loose fragments of stone on a west-facing slope below Robin Peak (60.6833° S, 45.6333° W) on Signy Island during the austral summer of 2015/16. All apparatus used for obtaining the samples was sterile and samples were stored and subsequently transported frozen (-20°C) in sterile containers to the Universiti Sains Malaysia. Cyanobacterial strains were then brought into culture by inoculation on 1% agarised full strength BG-11 medium in Petri dishes [45]. Culture media were supplemented with 100-µg mL^{-1} cycloheximide to prevent growth of eukaryotes [46]. Cultures were incubated at 15 ± 2°C with 24 h light supplied by cool white fluorescent lamps at 27 µmol m^{-2} s^{-1}. Light-limitation experiments were conducted at < 4 µmol m^{-2} s^{-1}. After 4 weeks of incubation, algal growth developing on the plates was examined microscopically.

Morphological characterization

Morphological examination was conducted using an Olympus BX-53 light microscope (Olympus America Inc., Center Valley, PA, USA) at 100 – 2000x magnification. Photomicrographs were taken. Illustrations were made with the aid of a *camera lucida*. Specimens were analysed based on morphological characteristics, particularly filament and trichome width, sheath morphology, cell colour, shape of intercalary and apical cells and presence of granules. Size measurements were made on 30 randomly chosen replicate

specimens for each morphospecies. The characteristics of the strain studied were compared with descriptions in the literature [8, 25, 26].

Transmission electron microscopy

Transmission electron microscopy (TEM) samples were fixed in McDowell-Trump fixative solution [47] prepared in 0.1 M phosphate buffer and later post-fixed with 1% osmium tetroxide. The fixed material was dehydrated in an ethanol series (50%, 75%, 95%, and 100%) and embedded in Spurr's resin [48] mixed in a rotator overnight. The ultrathin sections were treated with uranyl acetate and lead citrate to improve contrast [49]. Thin sections were collected on copper grids and were observed using a JEM 2000FX (JEOL, Tokyo, Japan) operating at 100 kV.

Molecular analyses

DNA was extracted using the G-spin for bacteria genomic DNA extraction kit (iNtRON Biotechnology, Korea) following the manufacturer's protocol. DNA sample concentration was measured using a Thermo Scientific NanoDrop instrument. The 16S rDNA gene and the 16S–23S internal transcribed spacer (ITS) were amplified using the polymerase chain reaction (PCR) and the combination of primers 2 (5' – GGG GGA TTT TCC GCA ATG GG – 3') and 3 (5' – CGC TCT ACC AAC TGA GCT A – 3') for the 16S rRNA gene and primers 1 (5'-CTC TGT GTG CCT AGG TAT CC – 3') and 5 (5' – TGT AGC TCA GGT GGT TAG – 3') for the ITS region [50]. This resulted in products of approximately 1 600 bp for the 16S rRNA gene and 600 bp for the ~~16S – 23S gene,~~ The reaction mix comprised 2 μL of extracted DNA used in 50 μL reactions containing 1 μL of each forward and reverse primer, 21 μL of ultrapure water and 25 μL of *MyTaq*™ *Red Mix*, which is a pre-prepared mixture of buffer, dNTPs and

Taq polymerase (Bioline, United Kingdom). PCR was carried out using a Bio-Rad Thermal Cycler with standard parameters set as follows: 95 °C for 2 min, 95 °C for 15 sec, 55 °C for 15 sec (30 cycles), and 7 min 20 sec extension at 72 °C. Once the reaction was completed, the integrity of the PCR product was verified using a 2 % agarose gel.

Phylogenetic analyses and ITS folding

All sequences were edited and assembled using the Geneious 11.0 software package (Biomatters, http://www. geneious.com). Sequence alignments were prepared using the MUSCLE algorithm in Geneious 11.0 and then manually checked by eye. The dataset included 47 OTUs, consisting of sequences newly obtained in this study together with additional closely related sequences of *Nodosilinea* species and other filamentous cyanobacteria taxa retrieved from GenBank, and one outgroup taxon (*Gloeobacter violaceus* FR798924). All new sequences generated in this study have been submitted to GenBank.

Phylogenetic analyses were performed using two different methods: maximum likelihood (ML) and Bayesian inference (BI). Before ML and BI analyses, the best-fit model of DNA substitution was determined with the program Kakusan4 [51]. ML analyses were performed using with RaxML v7 [52] in Geneious 11.0 using the general time-reversible invariant-sites (GTRI) nucleotide substitution model with the default parameters. The bootstrap probability of each branch was calculated using 1000 replications. BI analyses were performed with the program MrBayes v3.1.2 [53]. Two independent analyses, each consisting of four Markov chains, were run simultaneously for 3000000 generations, sampling every 100 generations. Log likelihood and parameter values were assessed with Tracers ver. 1.5 [54]. A burn-in of 25% of saved trees was removed, and the remaining trees were used to calculate the

Bayesian posterior probability values. ML and BI trees were edited with the program FigTree v1.3.1 [53].

The 16S–23S ITS region was used for modelling of secondary structure folding. The tRNA genes were identified using tRNAscan-SE 2 [55]. The secondary structure of the D1–D1´ helix was modelled using the Mfold WebServer with default conditions.

Results

The strain investigated shows the diagnostic traits of the genus *Nodosilinea*. However, it has characteristics that do not correspond to any previously described species. It is phylogenetically distinct within the genus with respect to the nucleotide sequences of both the 16S rDNA gene and 16S-23S ITS region. We propose the name *Nodosilinea signiensis* for this strain.

Class Cyanophyceae

Order Synechococcales

Family Leptolyngbyaceae

Genus Nodosilinea

Description. (Culture conditions) Mat creeping on agar, pale blue-green to olive-green in colour. Filaments long, immotile, solitary, occasionally forming spirals under normal light conditions of 27 µmol m^{-2} s^{-1} (Fig. 1 a, c). Under low light intensity of < 4 µmol m^{-2} s^{-1}, uniseriate trichomes can lie parallel or twisted around one another within a common sheath (Fig. 1 d, e). This resembles nodule formation whereby filament width in these areas becomes wider, up to 5.0 µm wide (Fig. 2 c - e). Cells 1.0 (1.5) – 2.0 µm wide, 1.0 – 2.0 (2.3) µm long, shorter to longer than wide, discoid to barrel-shaped (Fig. 2 a – g). Apical cells rounded, non–capitate, without calyptra and lacking granules (Fig. 2 g). Sheath colourless, thin.

Etymology. *Nodosilinea signiensis,* Nodosilinea = “Knotted line” Perkeson et al. (2011); *signiensis* (sig.nie’n.sis) adj. signiensis = originated from Signy Island.

Habitat. Mats in cracks and crevices in rocks and beneath loose fragments of stone collected from west-facing slope below Robin Peak (60.6833° S, 45.6333° W).

Occurrence. South Orkney Islands, Signy Island.

Observations. In cultures incubated for 4 weeks under low light, filaments are entangled, curved or very occasionally form a spiral. Cell division without lengthening of the enclosing sheath causes the trichomes sometimes bend and fragment to form two or three twisted structures that resemble nodules. This was rarely observed in the present strain.

Fig. 1. *Nodosilinea signiensis* grown in culture. a, filaments. **b**, rounded apical cell (arrow). **c**, spiral formation (arrow). **d & e**, trichomes twisted within the enclosing sheath, young trichome forming nodules (arrow). Scale bars: 20 µm.

Fig 2. Morphological characteristics of *Nodosilinea signiensis*. a – e, trichome variously curved, sometimes straight, tangled together or twisted, characteristic nodules (arrow). **f & g**, trichomes lying together within a common sheath, mature rounded apical cells (arrow). Scale bar: 10 µm.

Morphological assessment. The strain displays similar nodule formation to that of the seven currently described species within the genus. *Nodosilinea* sp. LEGE 13457 and *Nodosilinea* sp. TM-3.1 that originated from Antarctica [27] are not included here in the comparison as both specimens lack detailed taxonomic characterization. Cell width of *Nodosilinea signiensis* falls within the range of all previously recorded *Nodosilinea* species (Table 1). Cell length closely resembles five of the seven described species (*N. conica*, *N.*

chupicuarensis, *N. nodulosa*, *N. radiophila* and *N. ramsarensis*), while *N. epilithica* and *N. bijugata* have longer cells at 8 µm and 6.2 µm, respectively (Table 1). The vegetative cell shape of *N. signiensis* was distinct, being the only species showing a variety of cell shapes ranging from isodiametric to longer than wide or shorter than wide. Cell constriction changed from slightly constricted to distinctly constricted as trichomes matured. In all other *Nodosilinea* species, the cell shape was reported to be stable throughout the development of the trichome (Table 1). Trichomes of the present strain showed no attenuation towards the apex in contrast to the abrupt tapering in *N. conica* (Table 1). *N. signiensis* is the only species lacking cell granulation. Sheath development was very similar to *N. conica* and *N. chupicuarensis*, with the thin, colourless sheath occasionally becoming wide in mature filaments (Table 1). Three species, *N. conica*, *N. radiophila*, and *N. ramsarensis*, have been recorded from extreme environments (Table 1).

Table 1. Comparison of characteristics of the previously described eight species of *Nodosilinea* [8, 24, 25, 28] and *N. signiensis* sp. nov. ('?' indicates feature unknown).

**Characters**	***Nodosilinea signiensis***	***Nodosilinea epilithica***	***Nodosilinea bijugata***	***Nodosilinea conica***	***Nodosilinea chupicuarensis***	***Nodosilinea nodulosa***	***Nodosilinea radiophila***	***Nodosilinea ramsarensis***
**Cell length (µm)**	1.0 – 2.0 (2.3)	1.0 – 8.0	1.5 – 6.2	0.9 – 2.4	1.1 – 1.3	1.1 – 1.5	1.0 – 2.0	(0.8) 1.0 – 1.5
**Cell width (µm)**	1.0 (1.5)	1.5 – 2.5	1.5 – 1.7	2.5 – 2.7	1.2	1.2 – 2.4	2.0 – 5.0	1.0 – 2.0
**Cell shape**	Isodiametric, longer than wide/ barrel shape	Barrel shaped, shorter to longer than wide	Isodiametric, longer than wide	Isodiametric, shorter than wide	Isodiametric	Isodiametric, longer than wide	Isodiametric, longer than wide	Isodiametric, longer than wide
**Cross-wall**	Slightly constricted to strongly constricted	Distinctly constricted	Slightly constricted	Slightly constricted	Constricted	Slightly constricted to strongly constricted	Distinctly constricted	Distinctly constricted
**Filaments**	Solitary, immotile, forming spiral	Forming nodules in low light	Rarely forming nodules	Rarely forming nodules	Multiseriate, motile, forming nodules	Forming nodules	No formation of nodules	Rarely forming nodules
**Attenuated**	Absent	?	?	Tapering abruptly	?	?	?	?

**Apical cells**	Rounded	Rounded	Rounded	Rounded	Dome-shaped	Rounded	?	?
**Granule**	Ungranulated	Granulated	Granulated	?	Granulated at cross walls	Granulated at cross walls	Granulated at cross walls	Granulated
**Sheaths**	Very thin, colourless	Thin, colourless, occasionally becoming wide and diffluent	Often absent, thin, colourless	Soft, thin, colourless	Thin, clear	Thin, colourless, occasionally becoming wide and diffluent	Thin, colourless	Thin, colorless
**Special features**	Uniseriate trichome lie parallel or twisted around one another within a common sheath that resembles nodules	Cells typically barrel shaped after cell division, cylindrical in nondividing trichomes	Cells typically barrel shaped to spherical; inflated sheaths	Abundant nodule formation	Filament forming a tight spiral	?	?	?
**Occurrence**	Soil - Signy Island, Antarctica	House wall - Peninsula Gargano, town of Vieste (Foggia), Italy.	Littoral zone – Eutrophic Lake Piaseczno, Poland	Sevilleta Long Term Ecological Research, New Mexico Soil -Chihuahuan Desert, USA	Stone monument surface - Central Mexico	Marine - South China Sea	Benthic mat in thermal spring (27℃) - Talesh Mahalleh, Ramsar Iran	Soil around thermal spring (32℃) - Khaksefid, Ramsar, Iran.

Fig. 3. TEM of *Nodosilinea signiensis*. a, cross-section of a cell within its surrounding sheath. **b**, longitudinal section of a trichome. Scale bar: 1 µm. *s* = sheath; *t* = thylakoids; *n* = nucleoplasm. Thylakoids are arranged more or less parallel in a parietal position.

Transmission Electron Microscopy. Thylakoids were parietal (4-5 per cell), visible in longitudinal section but poorly seen under cross section (Fig 3). Nodule formation was not observed under TEM. Cyanophycin granules were visible among the thylakoids but carboxysomes were absent. The cell wall was simple, similar to *Leptolyngbya* species, and the sheath was distinct.

Phylogenetic analysis. The data set consisted of ~~1482~~ bp, including gaps. Partial sequences of the 16S rDNA of *N. signiensis* were distinct from other *Nodosilinea* sequences by at least 2% (29 bp). Both ML and BI analyses yielded an identical topology, and therefore only the ML tree with a – *ln* L score of 11654.762 is presented (Fig 4). The 16S rDNA phylogeny clearly indicated that the study strain was included in the major clade of *Nodosilinea* species (98% ML bootstrap percentage (BP) and 1.00 posterior probabilities (PP), which consists of 2 clades (N1 and N2). The relationships within the major *Nodosilinea* clade were more complex and showed several low to well supported clades. *N. signiensis* was clustered in clade N1, and formed a basal subclade, which presently contains nine sequences: six *Nodosilinea*, two "*Leptolyngbya*" and one "*Jaaginema*" (Fig 4). Within the basal clade, *N. signiensis* was sister to other species. The basal relationships were not well resolved.

Fig 4. Phylogenetic tree based on *Nodosilinea signiensis* 16S rRNA gene sequences. ML bootstrap values (left) followed by Bayesian posterior probabilities (right) on branches. Asterisks indicate full support, and dashes indicate support values less than 50%. The sequence generated in this study is in boldface. **N1** = *N. signiensis* sequences clustered with species from

the same and other current genera. **N2 =** distinct clade containing a separate *Nodosilinea* species. *Gleobacter violaceous* FR798924 was the outgroup.

Fig 5. Secondary structure based on 16S – 23S internal transcribed spacer (ITS). Eight *Nodosilinea* species. *N. signiensis* displayed identical structure with different sequence in every helix.

ITS Secondary structure. The D1–D1´ helix, a semi-conserved region of the 16S–23S ITS region, was analysed. The putative secondary structures of *N. signiensis*, together with other *Nodosilinea* species, are presented in Fig 5. The D1–D1´ helices of *N. signiensis* are identical with five previously recorded species: *N. radiophila* TMS2B, *N. ramsarensis* KH-S S2.6, *N. chupicuarensis* PCA471, *N. epilithica* Kovacik 1998/7, *N. nodulosa* UTEX 2910. Despite being identical in structure, every helix differed in sequence (Fig. 5). Comparison of secondary structure provides additional information in comparing the identity of the present strain with previous records.

Discussion

Our study on a cyanobacterial strain superficially resembling *Leptolyngbya,* isolated from soils in Signy Island, has resulted in the identification of a new species of *Nodosilinea*. *N. signiensis* is differentiated from other previously described species based on cell size, cell shape, filament attenuation, sheath morphology, granulation and geographical distribution. Due to the simple morphology of this genus, some characteristics overlap between the species. However, the distinct cell shapes and the lack of filament attenuation separates *N. signiensis*

from all previously described species. Cultivation showed that nodule formation is only facilitated in low light conditions. Therefore, strains assigned to similar genera but that have not been exposed to low light could have been misidentified. The occurrence of this species in an Antarctic terrestrial habitat further supports its separation from previously described species, based on the strikingly different biotope.

Our genetic data clearly indicate that *N. signiensis* is distinct from other *Nodosilinea* species. The 16S rDNA phylogenetic analysis confirms its position within the major clade of *Nodosilinea*. *N. signiensis* showed similarity values of lower than 98 % compared with species from the basal subclade within the clade N1. Stackbrandt and Goebel (1994) recommended that the similarity cut-off for bacterial species recognition should be 97% [56]. Recently, Stackbrandt and Ebers [57] raised the cut-off to 98.7%, a value later supported by Yarza et al. [58]. On this basis, *N. signiensis* is phylogenetically distinct from the seven previously described *Nodosilinea* species. However, the evolutionary relationships of *N. signiensis* within the genus remain unclear, possibly due to the limited number of variable sites in the 16S rDNA gene. Future use of multiple genetic makers will be required to clarify phylogenetic relationships within *Nodosilinea*.

Modelling of the secondary structure of the ITS region indicated that the structure of the D1-D1'region was identical, even though sequences were distinctly different to that of other *Nodosilinea* species. Previous studies have shown that analysis of 16S rDNA gene phylogeny coupled with the secondary structure of the ITS region provides a suitable tool for separation of cyanobacteria species [8, 59, 60, 61, 62]. This study confirms that the integration of morphological and genetic sequence data provides an effective means to validate the taxonomy of *Nodosilinea* species.

Acknowledgements

We thank Dr Japareng Lalung for collection of the original soil sample on Signy Island. The British Antarctic Survey staff at Signy research station are thanked for logistical and other practical support. This paper also contributes to the international SCAR 'State of the Antarctic Ecosystem' (AntEco) research programme.

References

1. Castenholz RW, Waterbury JB. Taxa of the cyanobacteria. Bergey's manual of systematic bacteriology. 1989; 3: 1727-1728.
2. Komárek J. A polyphasic approach for the taxonomy of cyanobacteria: principles and applications. European Journal of Phycology. 2016; 51: 346-353.
3. Komárek J, Kaštovský J, Mareš J, Johansen JR. Taxonomic classification of cyanoprokaryotes (cyanobacterial genera) using a polyphasic approach. Preslia. 2014; 86: 295-335.
4. Mareš J. Multilocus and SSU rRNA gene phylogenetic analyses of available cyanobacterial genomes, and their relation to the current taxonomic system. Hydrobiologia. 2018; 811: 19-34.
5. Komárek J. Several problems of the polyphasic approach in the modern cyanobacterial system. Hydrobiologia. 2018; 811:7-17.
6. Abed RM, Garcia-Pichel F, Hernández-Mariné M. Polyphasic characterization of benthic, moderately halophilic, moderately thermophilic cyanobacteria with very thin trichomes and the proposal of *Halomicronema excentricum* gen. nov., sp. nov. Archives of Microbiology. 2002; 177: 361-70.
7. Turicchia S, Ventura S, Komárková J, Komárek, J. Taxonomic evaluation of cyanobacterial microflora from alkaline marshes of northern Belize. 2. Diversity of oscillatorialean genera. Nova Hedwigia. 2009; 89: 165-200.
8. Perkerson III RB, Johansen JR, Kovácik L, Brand J, Kaštovský J, Casamatta DA. A unique Pseudanabaenalean (Cyanobacteria) genus *Nodosilinea* gen. nov. based on morphological and molecular data. Journal of Phycology. 2011; 47: 1397-1412.
9. Taton A, Wilmotte A, Šmarda J, Elster J, Komarek, J. *Plectolyngbya hodgsonii*: a novel filamentous cyanobacterium from Antarctic lakes. Polar Biology. 2011; 34: 181-191.

10. Dadheech PK, Mahmoud H, Kotut K, Krienitz L. *Haloleptolyngbya alcalis* gen. et sp. nov., a new filamentous cyanobacterium from the soda lake Nakuru, Kenya. Hydrobiologia. 2012; 691: 269-283.

11. Zammit G, Billi D, Albertano P. The subaerophytic cyanobacterium *Oculatella subterranea* (Oscillatoriales, Cyanophyceae) gen. sp. nov.: a cytomorphological and molecular description. European Journal of Phycology. 2012; 47: 341-354.

12. Dvořák PE, Hindák FR, Hašler PE, Hindakova A, Poulíčková AL. Morphological and molecular studies of *Neosynechococcus sphagnicola*, gen. et sp. nov. (Cyanobacteria, Synechococcales). Phytotaxa. 2014; 170: 24-34.

13. Vaz MG, Genuario DB, Andreote AP, Malone CF, Sant'Anna CL, Barbiero L, Fiore MF. *Pantanalinema* gen. nov. and *Alkalinema* gen. nov.: novel pseudanabaenacean genera (Cyanobacteria) isolated from saline–alkaline lakes. International Journal of Systematic and Evolutionary Microbiology. 2015; 65: 298-308.

14. Song G, Jiang Y, Li R. *Scytolyngbya timoleontis*, gen. et sp. nov. (Leptolyngbyaceae, Cyanobacteria): a novel false branching Cyanobacteria from China. Phytotaxa, 2015; 224(1): 72-84.

15. Dvořák P, Hašler P, Pitelková P, Tabakova P, Casamatta, DA, Poulíčková A. A new cyanobacterium from the Everglades, Florida–*Chamaethrix* gen. nov. Fottea. 2017; 17: 269-276.

16. Li X, Li R. *Limnolyngbya circumcreta* gen. & comb. nov. (Synechococcales, Cyanobacteria) with three geographical (provincial) genotypes in China. Phycologia. 2016; 55: 478-491.

17. Sciuto K, Moro I. Detection of the new cosmopolitan genus *Thermoleptolyngbya* (Cyanobacteria, Leptolyngbyaceae) using the 16S rRNA gene and 16S–23S ITS region. Molecular Phylogenetics and Evolution. 2016; 105: 15-35.

18. Miscoe LH, Johansen JR, Kociolek JP, Lowe RL, Vaccarino MA, Pietrasiak N, Sherwood AR. The diatom flora and cyanobacteria from caves on Kauai, Hawaii. Acta Botanica Hungarica. 2016; 58: 3-4.

19. Dvořák P, Hašler P, Pitelková P, Tabáková P, Casamatta DA, Poulíčková A. A new cyanobacterium from the Everglades, Florida—*Chamaethrix* gen. nov. Fottea, Olomouc. 2017; 17: 269-76.

20. Sciuto K, Moschin E, Moro I (2017). Cryptic cyanobacterial diversity in the Giant Cave (Trieste, Italy): the new genus *Timaviella* (Leptolyngbyaceae). Cryptogamie, Algologie. 2017; 38(4): 285-324.

21. Jahodářová E, Dvořák P, Hašler P, Poulíčková A. Revealing hidden diversity among tropical cyanobacteria: the new genus *Onodrimia* (Synechococcales, Cyanobacteria) described using the polyphasic approach. Phytotaxa. 2017; 326: 28-40.

22. Brito A, Ramos V, Mota R, Lima S, Santos A, Vieira J, Tamagnini, P. Description of new genera and species of marine cyanobacteria from the Portuguese Atlantic coast. Molecular Phylogenetics and Evolution. 2017; 111: 18-34.

23. Zimba PV, Huang IS, Foley JE, Linton EW. Identification of a new- to- science cyanobacterium, *Toxifilum mysidocida* gen. nov. & sp. nov. (Cyanobacteria, Cyanophyceae). Journal of Phycology. 2017; 53: 188-197.

24. Li Z, Brand J. *Leptolyngbya nodulosa* sp. nov. (Oscillatoriaceae), a subtropical marine cyanobacterium that produces a unique multicellular structure. Phycologia. 2007; 46: 396-401.

25. Vazquez-Martinez J, Gutierrez-Villagomez JM, Fonseca-Garcia C, Ramirez-Chavez E, Mondragón-Sánchez M L, Partida-Martinez L, Molina-Torres J. *Nodosilinea chupicuarensis* sp. nov. (Leptolyngbyaceae, Synechococcales) a subaerial cyanobacterium isolated from a stone monument in central Mexico. Phytotaxa. 2018; 334: 167-182.

26. Anagnostidis K, Komarek J. Cyanoprokariota. Teil 2: Oscillatoriales. *SuBwasserflora von Mitteleuropa*; Band 19/2 Spektrum Akademischer Verlag Heidelberg. 2005.

27. Costa MS, Rego A, Ramos V, Afonso TB, Freitas S, Preto M, Lopes V, Vasconcelos V, Magalhaes C, Leao PN. The conifer biomarkers dehydroabietic and abietic acids are widespread in Cyanobacteria. Scientific Reports. 2016; 6: 23436.

28. Heidari F, Hauer T, Zima J, Riahi H. New simple trichal cyanobacterial taxa isolated from radioactive thermal springs. Fottea. 2018; 18:137-49.

29. Kongisser RA. On the Methods of Studying Phytoplankton [In Russian]. Trav. Soc. Nat. Leningrad. 1925.

30. Wynn-Williams DD. 3.5 Television image analysis of microbial communities in Antarctic fellfields. Polarforschung. 1988; 58: 239-249.

31. Gaydon DS, Probert ME, Buresh RJ, Meinke H, Timsina J. Modelling the role of algae in rice crop nutrition and soil organic carbon maintenance. European Journal of Agronomy. 2012; 39: 35-43.

32. Friedmann EI. Endolithic microorganisms in the Antarctic cold desert. Science. 1982; 215: 1045-53.

33. Quesada A, Vincent WF (2012). Cyanobacteria in the cryosphere: snow, ice and extreme cold. In Ecology of cyanobacteria II. Dordrecht: Springer; 2012. pp. 387-399.

34. Broady PA. The terrestrial algae of Signy Island, South Orkney Islands. British Antarctic Survey; 1979.

35. Friedmann EI, Hua M, Ocampo-Friedmann R. Cryptoendolithic lichen and cyanobacterial communities of the Ross Desert, Antarctica. Polarforschung. 1988; 58: 251-259.

36. Priscu JC, Fritsen CH, Adams EE, Giovannoni SJ, Paerl HW, McKay CP, Doran PT, Gordon DA. Lanoil BD, Pinckney JL. Perennial Antarctic lake ice: an oasis for life in a polar desert. Science. 1998; 280: 2095-2098.

37. Mataloni G, Tell G, Wynn-Williams DD. Structure and diversity of soil algal communities from Cierva Point (Antarctic Peninsula). Polar Biology. 2000; 23: 205-211.

38. Cavacini, P. Soil algae from northern Victoria Land (Antarctica). Polar Bioscience 2001; 14: 45-60.

39. Mueller DR, Pollard WH. Gradient analysis of cryoconite ecosystems from two polar glaciers. Polar Biology. 2004; 27: 66-74.

40. Brinkmann M, Pearce DA, Convey P, Ott, S. The cyanobacterial community of polygon soils at an inland Antarctic nunatak. Polar Biology. 2007; 30: 1505-1511.

41. Vishnivetskaya TA (2009). Viable cyanobacteria and green algae from the permafrost darkness. In Permafrost soils. Berlin, Heidelberg: Springer; 2009. pp. 73-84.

42. Taton A, Grubisic S, Brambilla E, De Wit R, Wilmotte A. Cyanobacterial diversity in natural and artificial microbial mats of Lake Fryxell (McMurdo Dry Valleys, Antarctica): a morphological and molecular approach. Applied and Environmental Microbiology. 2003; 69: 5157-69.

43. Stoyanov P, Moten D, Mladenov R, Dzhambazov B, Teneva I. Phylogenetic relationships of some filamentous cyanoprokaryotic species. Evolutionary Bioinformatics. 2014; EBO-S13748.

44. Jancusova M, Kovacik L, Pereira AB, Dusinsky R, Wilmotte A. Polyphasic characterization of 10 selected ecologically relevant filamentous cyanobacterial strains from the South Shetland Islands, Maritime Antarctica. FEMS Microbiology Ecology. 2016; 92: fiw100.

45. Rippka R, Deruelles J, Waterbury JB, Herdman M, Stanier RY. Generic assignments, strain histories and properties of pure cultures of cyanobacteria. Microbiology. 1979; 111: 1-61.

46. Bolch CJ, Blackburn SI. Isolation and purification of Australian isolates of the toxic cyanobacterium *Microcystis aeruginosa* Kütz. Journal of Applied Phycology. 1996; 8: 5-13.

47. McDowell EM, Trump BF. Histologic fixatives suitable for diagnostic light and electron microscopy. Archives of pathology & laboratory medicine. 1976; 100: 405-414.

48. Spurr AR. A low-viscosity epoxy resin-embedding medium for electron microscopy. Journal of ultrastructure research. 1969; 26: 31-43.

49. Reynolds ES. The use of lead citrate at high pH as an electron-opaque stain in electron microscopy. The Journal of cell biology. 1963; 17: 208.

50. Boyer SL, Flechtner VR, Johansen JR. Is the 16S–23S rRNA internal transcribed spacer region a good tool for use in molecular systematics and population genetics? A case study in cyanobacteria. Molecular Biology and Evolution. 2001; 18: 1057-1069.

51. Kanehisa M, Goto S, Sato Y, Furumichi M, Tanabe M. KEGG for integration and interpretation of large-scale molecular data sets. Nucleic Acids Research. 2011; 40: D109-D114.

52. Stamatakis A. RAxML-VI-HPC: maximum likelihood-based phylogenetic analyses with thousands of taxa and mixed models. Bioinformatics. 2006; 22: 2688-2690.

53. Ronquist F, Huelsenbeck JP. MrBayes 3: Bayesian phylogenetic inference under mixed models. Bioinformatics. 2003; 19: 1572- 1574.

54. Rambaut A. FigTree, version 1.3. 1. Computer program distributed by the author; 2009, http://treebioedacuk/software/figtree.

55. Lowe TM, Chan PP. tRNAscan-SE On-line: integrating search and context for analysis of transfer RNA genes. Nucleic Acids Research. 2016; 44: W54-7.

56. Stackebrandt E, Goebel BM. Taxonomic note: a place for DNA-DNA reassociation and 16S rRNA sequence analysis in the present species definition in bacteriology. International Journal of Systematic Evolutionary Microbiology. 1994; 44: 846-849.

57. Stackebrandt E. Taxonomic parameters revisited tarnished gold standards. Microbiology Today. 2006; 33: 152-155.

58. Yarza P, Yilmaz P, Pruesse E, Glöckner FO, Ludwig W, Schleifer KH, Rosselló-Móra R. Uniting the classification of cultured and uncultured bacteria and archaea using 16S rRNA gene sequences. Nature Reviews Microbiology. 2014; 12: 635.

59. Řeháková K, Johansen JR, Casamatta DA, Xuesong L, Vincent J. Morphological and molecular characterization of selected desert soil cyanobacteria: three species new to science including *Mojavia pulchra* gen. et sp. nov. Phycologia. 2007; 46: 481-502.

60. Johansen JR, Kovacik L, Casamatta DA, Iková KF, Kaštovský J. Utility of 16S-23S ITS sequence and secondary structure for recognition of intrageneric and intergeneric limits within cyanobacterial taxa: *Leptolyngbya corticola* sp. nov. (Pseudanabaenaceae, Cyanobacteria). Nova Hedwigia. 2011; 92: 283-302.

61. Vaccarino MA, Johansen JR. *Brasilonema angustatum* sp. nov (Nostocales), a new filamentous cyanobacterial species fom the Hawaiian Islands. Journal of Phycology. 2012; 48(5): 1178-1186.

62. Osorio-Santos K, Pietrasiak N, Bohunická M, Miscoe LH, Kováčik L, Martin MP, Johansen JR. Seven new species of *Oculatella* (Pseudanabaenales, Cyanobacteria): taxonomically recognizing cryptic diversification. European Journal of Phycology. 2014; 49(4): 450-470.

Figure 1 Click here to download Figure Fig. 1.tif

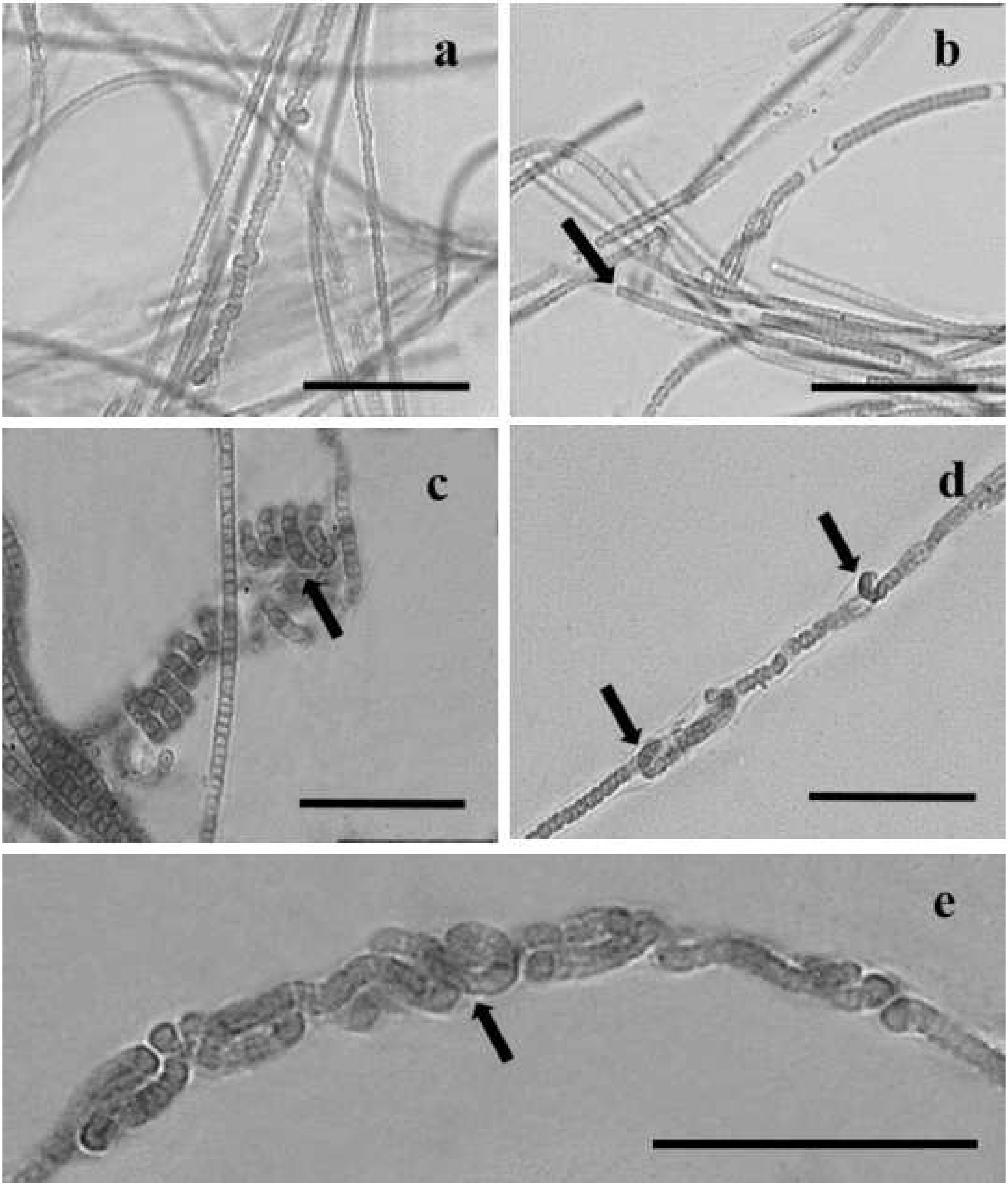

Figure 2

Click here to download Figure Fig. 2.tif

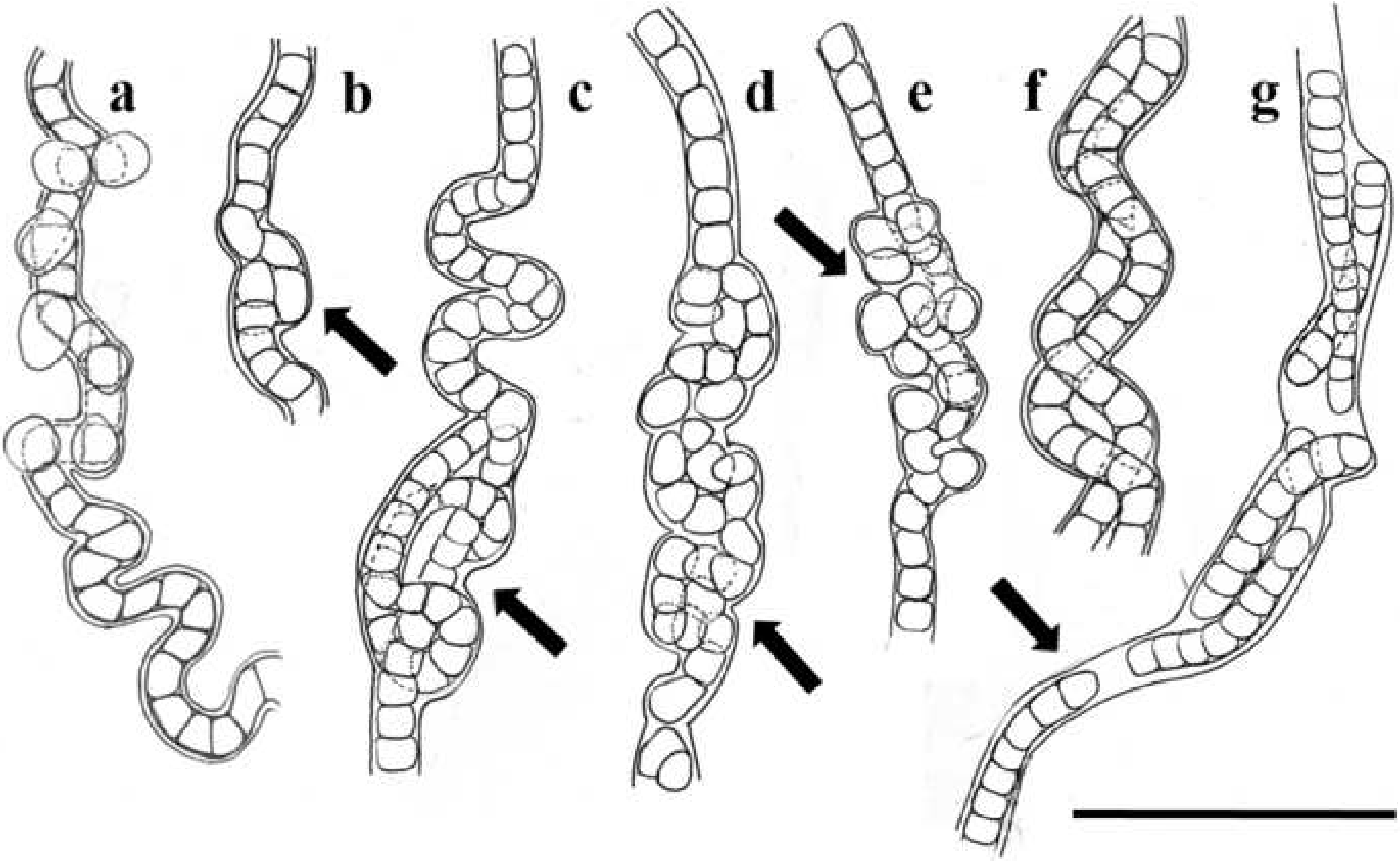

Figure 3

Click here to download Figure Fig. 3.tif

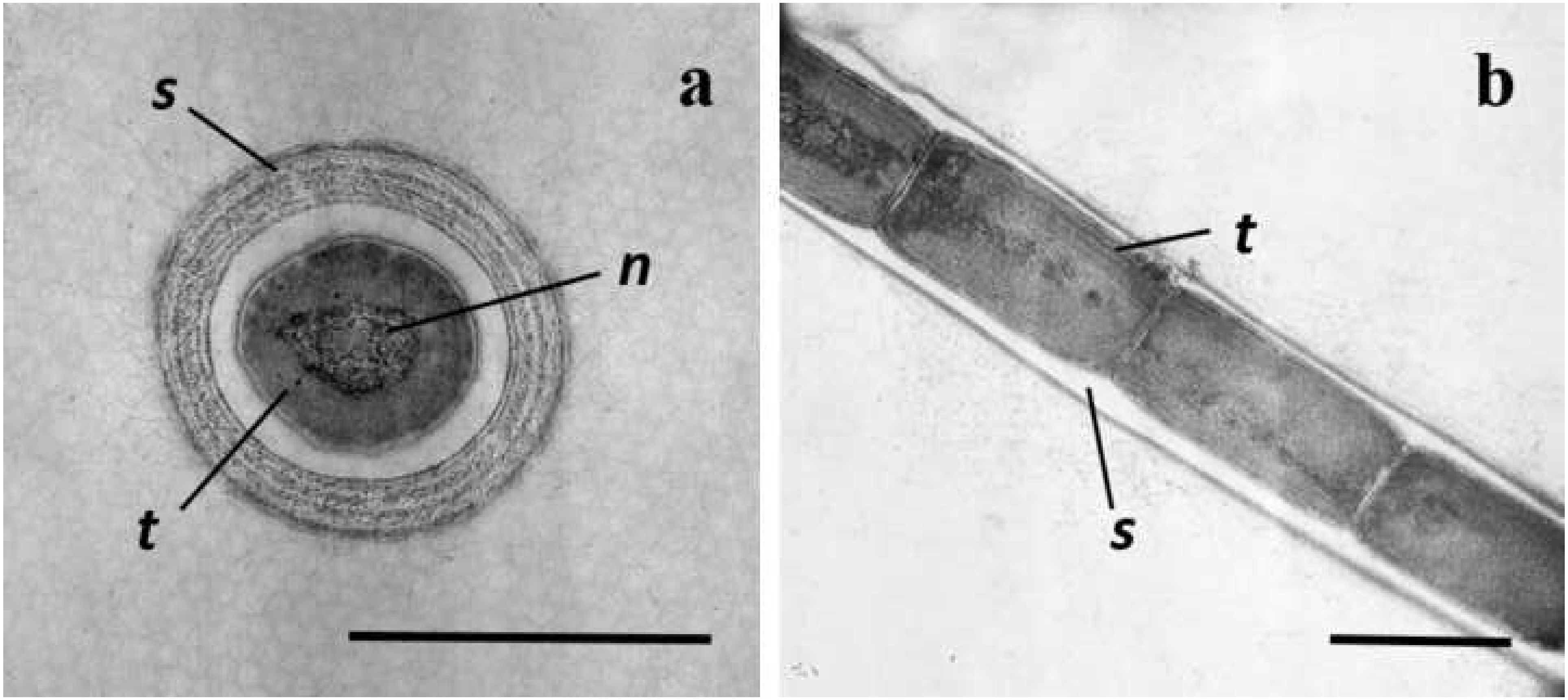

Figure 4

Click here to download Figure Fig. 4.tif

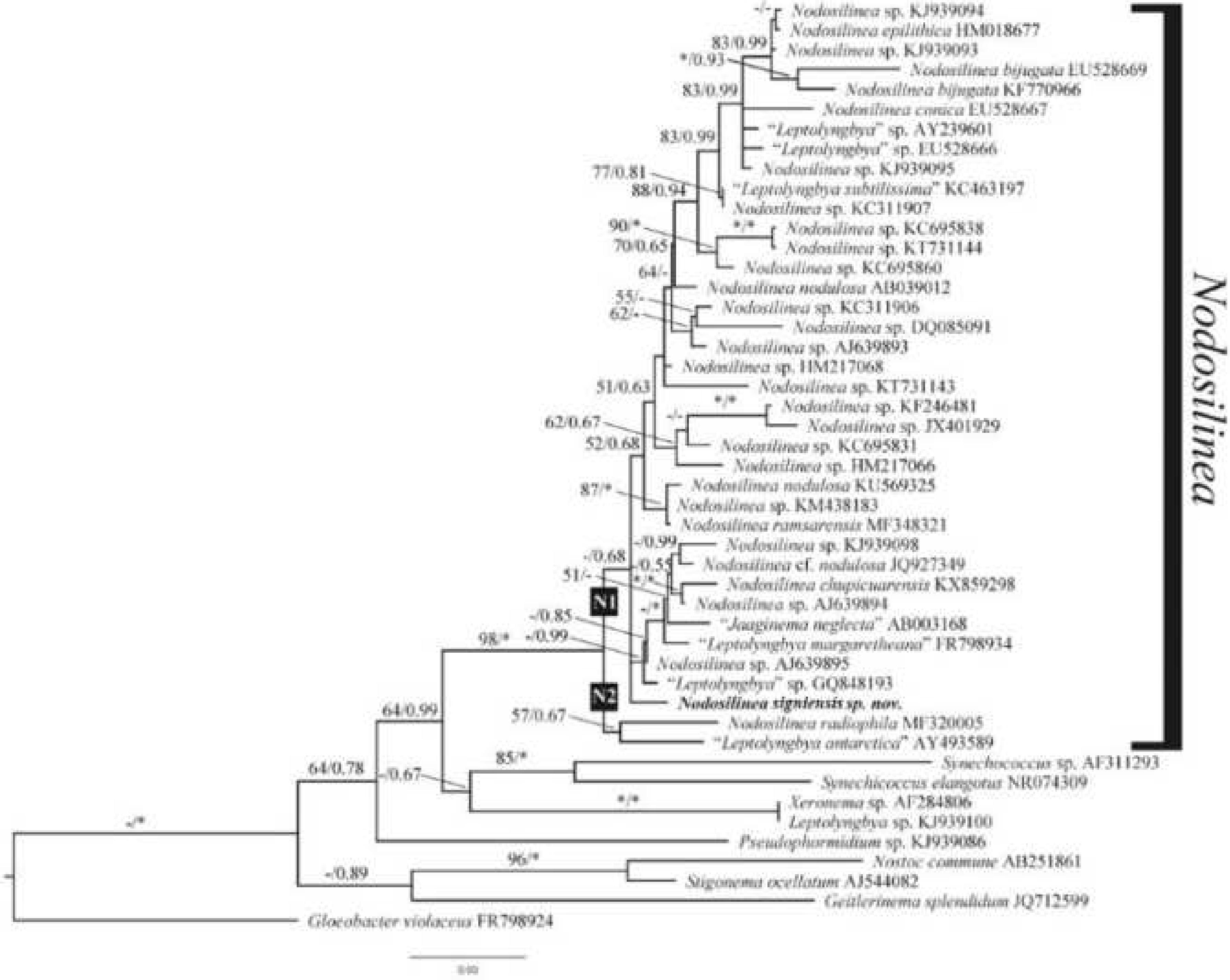

Figure 5

Click here to download Figure Fig. 5.tif

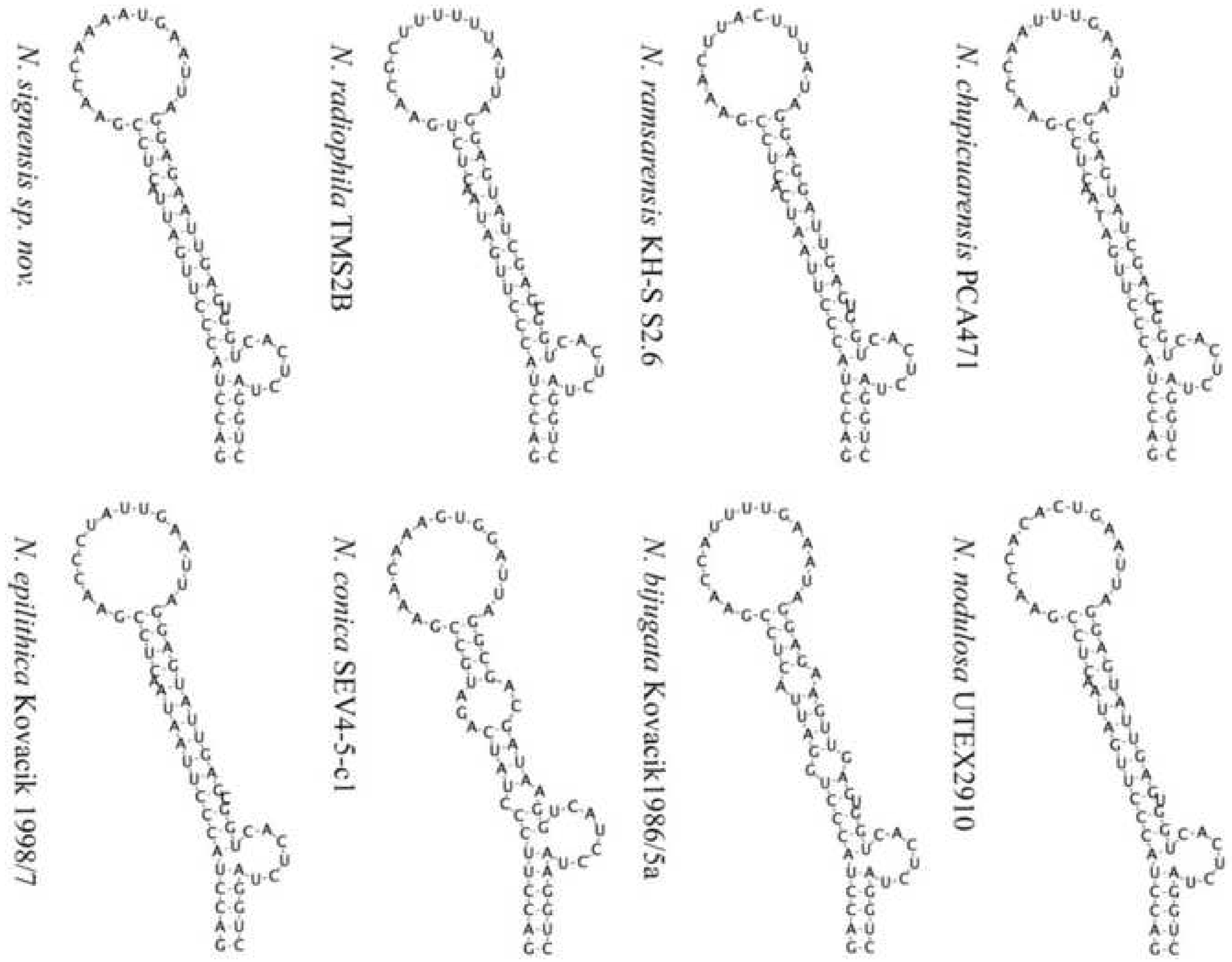

Supplement: Attachment — Submitted filename: PONE-D-19-15186 -reviewed.pdf [file pone.0224395.s001.pdf]
